# Endorsing a Civic (vs. an Ethnic) Definition of Citizenship Predicts Higher Pro-minority and Lower Pro-majority Collective Action Intentions

**DOI:** 10.3389/fpsyg.2018.01402

**Published:** 2018-08-07

**Authors:** Anna Kende, Nóra A. Lantos, Péter Krekó

**Affiliations:** ^1^Social Groups and Media Research Lab, Department of Social Psychology, Eötvös Loránd University, Budapest, Hungary; ^2^Doctoral School of Psychology, Eötvös Loránd University, Budapest, Hungary

**Keywords:** citizenship, ethnic, civic, collective action, Roma, immigrants

## Abstract

Europe has witnessed a polarization of intergroup attitudes and action tendencies in the context of the refugee crisis of 2015 and the rise of right-wing populism. Participation in both pro-minority collective action and right-wing nationalist movements has increased among members of ethnic majority groups. We analyzed these collective action intentions toward Roma people and Muslim immigrants in Hungary related to concepts of citizenship. In an online survey relying on a probabilistic sample that is demographically similar to the Hungarian population (*N* = 1069), we tested whether relying on the concept of ethnic citizenship predicted higher intentions to engage in pro-majority collective action, and lower intentions to engage in pro-minority collective action, and whether the connection was mediated by fear and empathy. We expected that the connections would be the opposite for civic citizenship. Our results supported the hypotheses, but we found that the ethnic definition was a stronger predictor of intergroup action intentions toward the immigrant group, and the civic definition a stronger predictor in case of the Roma minority group. In a second study (*N* = 320) we collected experimental evidence to show that civic and ethnic citizenship affected both types of collective action tendencies. We found that the manipulation had an effect on the concept of citizenship only in the ethnic dimension. Nevertheless, it influenced pro-minority collective action intentions especially in the presence of high empathy and low fear in the expected direction, that is, pro-minority collective action intentions were higher in the civic citizenship condition than in the ethnic citizenship condition. The effect was not found with regard to pro-majority collective action intentions. These findings highlight the potential consequences of nationalist rhetoric on intergroup action intentions and point out both the scope and the limits of influencing its effect.

## Introduction

The way in which high status, privileged, ethnic majority members of society act toward minorities has a huge impact on the situation of disadvantaged groups, and on society as a whole. In recent years we witnessed a rise in volunteerism and various forms of pro-social behavior toward members of disadvantaged minority groups, such as refugees (see e.g., Hamann and Karakayali, 2016). At the same time, there has also been a rise in intolerance (Pew Research, 2016), open hostility and hate crimes (Williamson, 2016; Pew Research, 2017), and protests of nationalist white supremacy groups both in Europe and in the United States (Muis and Immerzeel, 2017). These changes can be explained by the increasing acceptability of these attitudes and behaviors (Crandall et al., 2018). The aim of the current study is to understand how different definitions of the nation can predict and influence intergroup behavioral intentions of majority group members toward minorities, specifically with regard to the Roma ethnic minority group and Muslim immigrants in Hungary. We consider this issue particularly important in the current social and political context of Europe where Roma people are treated as second rate citizens, and terrorist attacks by Islamic extremists have almost all been committed by second or third generation immigrants, pointing to substantial problems with the social inclusion of immigrants in the national ingroup.

### Definitions of the Nation

The nation can be defined as an imagined community (Anderson, 1983) with shared history, culture, and laws (Smith, 1991). There are two chief mechanisms that describe the conditions of membership in the national ingroup, generally referred to as ethnic vs. civic definitions of citizenship (Kohn, 1944; Brubaker, 1996). Although the actual content of civic vs. ethnic definitions varies historically and across countries, there is ample empirical evidence for the continuing validity of this distinction despite the fact that the legal systems of all countries include both definitions to some extent. This evidence suggests that an ethnic definition of citizenship considers ancestry as the most important criterion of inclusion (i.e., *jus sanguinis* or the right of blood). An ethnic definition also entails that members of the nation have a common cultural heritage, language, and religion, and the group can be identified unambiguously (Smith, 1991). In contrast, a civic definition entails that citizenship can be gained by efforts to join the group and adherence to legal norms (Reeskens and Hooghe, 2010). The civic definition of citizenship can be exemplified by the concept of French nationhood that is strongly connected to the ideals of the French revolution resulting in France becoming a melting pot of nations with a disregard of ethnicity (see Berdah, 2006). It does not follow that legal requirements are not important for ethnic citizenship, it simply indicates that legal compliance and efforts are not sufficient. This expectation of legal adherence within both citizenship concepts implies that the two approaches are not entirely antagonistic, and tend to correlate positively rather than negatively (see Pehrson et al., 2009b; Reeskens and Hooghe, 2010). Endorsement of either of the two definitions of citizenship can be grasped on an individual level and on macro or cultural levels. Members of ethnically and culturally relatively homogenous countries are more likely to rely on ethnic definitions than civic ones (Reeskens and Hooghe, 2010).

These two approaches to citizenship have entirely different consequences for immigrants and members of ethnic minority groups. The concept of ethnic citizenship denies acceptance into the national ingroup for people who are ethnically different, while civic citizenship permits the acceptance of those who comply to the legal requirements of being or becoming a citizen. Therefore, a predominantly ethnic definition of citizenship can lead to higher hostility toward non-ethnic immigrants (Pehrson et al., 2009b; Reijerse et al., 2012; Verkuyten and Martinovic, 2015; Mepham and Verkuyten, 2017), and within the framework of ethnic citizenship the connection between national identity and prejudice against non-members is stronger (see Örkény, 2005; Pehrson et al., 2009a). Politicians often rely on this connection and use the concept of ethnic citizenship or some variation of it to promote anti-immigrant policies. This has been the case with most European right-wing populist parties that built their support by presenting immigrants as an ethnic threat (see Lucassen and Lubbers, 2012), but perhaps a better-known example is the anti-immigrant rhetoric used by Trump’s 2016 presidential campaign focusing on American national identity (see Knowles and Tropp, 2018).

However, some minority groups are closer to the ingroup than others because of a shared history or smaller perceived cultural distance. Therefore, traditional ethnic minority groups with a shared history may be perceived as less distant than ethnically different, new immigrant groups (Parker, 2010). This is important because perceived intergroup distance can have consequences for moral obligations toward members of the outgroup (Coryn and Borshuk, 2006; Hadarics and Kende, 2018), and high perceived distance limits their potential inclusion. Thus, both the definition of citizenship and perceived intergroup distance can influence whether ethnic majority citizens consider members of immigrant and ethnic minority groups as potential citizens.

Besides different definitions of citizenship and consequently who is included in or excluded from the national ingroup, we can also distinguish between different modes of identification with the nation. Roccas et al. (2006) used the terms attachment and glorification to label two distinct psychological mechanisms of group identification. People feel attachment with their ingroup if the group merely represents a source of positive self-esteem in line with the original claims of the social identity theory (Tajfel and Turner, 1979). Glorification, on the other hand, means that members consider their group superior to other groups resulting in uncritical loyalty to the group by its members. These two modes of identification are manifested in different forms of national identity and reflected in the distinctions between patriotism and nationalism (see Li and Brewer, 2004; Wagner et al., 2012; Heinrich, 2016), genuine patriotism and pseudo-patriotism (Adorno et al., 1950), and blind and constructive patriotism (Schatz et al., 1999). These terms all reveal a distinction between one’s positive emotional tie to the nation and the uncritical belief in its superiority.

Predictably, these two modes of identification with the nation have different consequences for attitudes toward integration of minorities and toward immigration in general (for an overview see Huddy, 2016). There is ample empirical evidence that blind/pseudo-patriotism or nationalism is associated with higher xenophobia (Spry and Hornsey, 2007), and lead to the escalation of intergroup conflicts through increased cognitive bias, stereotyping, moral disengagement (Leidner et al., 2010; Dugas et al., 2017), and sensitivity to threat and provocation (Sahar, 2008; Leidner and Castano, 2012; Steele et al., 2015; Rovenpor et al., 2016). Research has also shown that nationalism is more systematically associated with outgroup derogation than national attachment or constructive patriotism (for a review see Golec de Zavala et al., 2017).

Evidence for the connection between nationalism and intergroup hostility is straightforward. However, it has been more difficult to demonstrate that attachment with the ingroup or constructive patriotism is associated with positive rather than negative attitudes toward immigrants and ethnic minorities (Parker, 2010). The difficulty of establishing the connection between genuine patriotism and positive outgroup attitudes has at least three key reasons. Firstly, it has to do with the basic assumptions of social identity theory that suggests an inherent need for positively differentiating the ingroup even at the cost of discriminating against the outgroup (Tajfel and Turner, 1979). Secondly, it can be explained by the association between all forms of positive attitudes toward the national ingroup and right-wing or conservative political ideologies (see e.g., Duckitt and Sibley, 2016; with the exception of constructive patriotism, the operationalization of which includes the endorsement of social change efforts and shows no association with political ideology or political party identification, see Schatz et al., 1999). And thirdly, this difficulty may be explained by different outcomes of genuine patriotism toward different types of outgroups. According to Parker (2010) study, blind patriotism of US respondents predicted hostility toward a number of outgroups, such as African Americans, Jews, and Arabs, but symbolic patriotism promoted positive rather than negative attitudes mostly toward “domestic out-groups.” Thus, the positive association was much weaker for Arab people than for African Americans or Jews. Nevertheless, there is evidence that positive identification with the nation can increase positive attitudes toward immigration by, for example, emphasizing its inclusive character (Minescu et al., 2008; Wagner et al., 2012). In summary, nationalism is associated with the derogation of outgroups, such as immigrants and ethnic minorities, while at least some forms of patriotism can function as a protection from these forms of hostilities.

Blind patriotism or nationalism does not simply reflect a mode of identification, it also implies an essentialist view of the ingroup (Leyens et al., 2003). Therefore, ethnic citizenship is closely associated with nationalism, having similar consequences in terms of attitudes toward non-ethnic immigrants and ethnic minority groups. Schmidt et al. (2016) showed that higher endorsement of the ethnic definition of citizenship predicted higher level of exclusion from rights of non-ethnic migrants in Germany while this connection was not found with the civic definition. Sides and Citrin (2007) found that insistence on cultural unity (i.e., belief in a culturally homogenous concept of the nation) was the most robust predictor of anti-immigrant attitudes in the 15 EU member states of that time and in Hungary, the Czechia, and Poland based on the 2002–2003 European Social Survey data. Mepham and Verkuyten (2017) found that civic as opposed to ethnic concepts of citizenship predicted greater support for immigrant rights, and this relationship was mediated by perceived indispensability of immigrant groups for the ingroup. These results suggest that personal endorsement of ethnic citizenship and nationalism both have similarly negative consequences for intergroup attitudes, and personal endorsement of civic citizenship and genuine patriotism both have similarly positive consequences.

Intergroup attitudes of advantaged, majority group members toward minority groups are undeniably important and central elements of intergroup relations. Openly hostile attitudes can lead to discrimination or the rejection of ally activism (Çakal et al., 2016), and more subtle forms of prejudice can create obstacles to recognizing intergroup injustices (Powell et al., 2005; Becker and Wright, 2011; Case et al., 2014). However, general attitudes such as prejudice are not very accurate predictors of actual behavior. For this reason, we analyzed the influence of different definitions of citizenship on behavioral intentions that are better predictors of behavior than attitudes (in line with the theory of reasoned action by Ajzen and Fishbein, 1980, and the theory of planned behavior by Ajzen, 1991) by measuring pro-minority and pro-majority collective action intentions rather than intergroup attitudes. While there is a well-known gap between behavior intentions and actual behavior that should be taken into considerations (Sheeran’s, 2002 review revealed that intention strength can predict 28% of the variance of actual behavior), expressing intentions to engage in certain type of intergroup behavior can also influence social norms of behavior. These norms are in turn a strong predictor of actual behavior (see Cialdini et al., 1990; Nolan et al., 2008). Therefore, the point of studying behavior intentions is not simply related to the fact that these intentions may be realized as actions, but also to the fact that they can serve as descriptive norms of behavior for others. Research on collective action clearly suggests that actual participation is strongly influenced by the perceived behavior intention of others (e.g., Bolsen et al., 2014). This fits into our interest in social movements, such as the politically antagonistic pro-minority movements and right-wing nationalist movements that shape intergroup relations in Europe.

### Motivations for Pro-majority and Pro-minority Collective Action

People engage in collective action to escape a negative social identity by improving the intergroup situation for the benefit of their group (Tajfel and Turner, 1979; Wright et al., 1990), especially if the group suffers from unjust disadvantages (van Zomeren et al., 2011). Consequently, collective action signals the social change efforts to eliminate threats to the positive identity of the ingroup. It can be considered a form of social competition that members of groups engage in against those individuals, groups, or authorities whom they identify as responsible for the unjust intergroup situation (Simon and Klandermans, 2001).

Collective action research has been primarily concerned with and informed by progressive movements of minority groups and civil right movements. For this reason, pro-majority collective action (i.e., populist radical right movements, white supremacy movements, or extreme right-wing movements) have fallen outside the scope of social psychological research on collective action. However, both structurally disadvantaged and advantaged members of society can experience that their group was treated unfairly, in an unjust way, or it is affected by relative deprivation (see Runciman, 1966). Consequently, members of the majority can also develop intentions to engage in collective action based on similar psychological motivations to improve the situation of their ingroup regardless of their otherwise advantaged position in society (Leach et al., 2007).

In fact, white supremacy or populist majority movements may not be that different from for example civil rights movements when they demand equal rights for white people, the restoration of perceived injustices, and refer to disadvantages suffered by the majority group (Blee and Creasap, 2010). Nevertheless, this form of collective action was mostly examined within research on right-wing extremism focusing on the individual psychology of followers (in line with Adorno et al., 1950; see e.g., Simi et al., 2017), and explained by right-wing authoritarianism (RWA, Altemeyer, 1988), the role of psychological distress and experience of threat (e.g., Canetti-Nisim et al., 2009), and bias in social cognition, such as processing of fake news and information on conspiracies (Van Prooijen et al., 2015). While these approaches provide valuable insights into individual differences in the appeal of nationalist, pro-majority movements, they overlook group processes that may be more similar in both advantaged and disadvantaged groups. Putting together the argument of social identity theory (Tajfel and Turner, 1979), and the special characteristics of followers of right-wing extremist movements, we can conclude that nationalist, pro-majority social movements emerge as a result of perceived injustices and relative deprivation experienced by those members of the majority group who are especially sensitive to identity threats by for example the presence and influence of minority groups and immigration.

The interconnectedness of nationalism, perceived threat, and intergroup hostility has been shown by research conducted following terrorist attacks (e.g., Slone, 2000; Coryn et al., 2004), and in more stable situations as well. Perceived threat to the nation was found to increase xenophobic attitudes especially among people who already identified strongly with the nation (Sniderman et al., 2004). However, this is not a one-way process, threat does not only increase hostility toward outgroups, but it also strengthens national identification and therefore contributes to the vicious circle of conflict escalation (see Blank and Schmidt, 2003). This circular connection suggests that nationalism may increase threat perceptions related to outgroups, and at the same time the connection between nationalism and intergroup hostility may be increased in the presence of threat. These different processes highlight that while it may be more meaningful to conceptualize the role of threat as a mediator in the process, it can also be considered a moderator. In summary, members of the majority ethnic group may show higher intentions to engage in pro-majority collective action if they feel that their national identity is threatened by outgroups (in line with Mudde, 2004; Hirsch-Hoefler et al., 2010). Furthermore, perceived threat related to ethnic minorities or non-ethnic immigrants may be especially high among people who endorse the more essentialized and fixed ethnic rather than the more flexible civic definition of citizenship.

Intergroup hostility and nationalist movements against minorities are only one side of the coin though, and do not grasp the political and social context of contemporary intergroup issues in its entirety. The same situations that evoke fear among some people, evoke empathy in others. For example, following the 9/11 terrorist attacks, some members of the majority white population in the United States pleaded for racial tolerance and condemned the vicarious retaliation against Muslim people and immigrants (Reed and Aquino, 2003). When people feel empathy with victims of injustice, they recognize their suffering and feel motivated to engage in collective action as if they experienced injustices on behalf of their own ingroup (Thomas et al., 2009; Saab et al., 2015). Majority group members can feel empathy and become aware of social injustices suffered by members of an out-group as a result of intergroup contact (Selvanathan et al., 2017) or because these injustices violate their own moral principles. This recognition motivates people to eliminate the violation through politicized identification with the relevant ingroup (van Zomeren et al., 2012). The relevant ingroup may be an opinion-based group which provides a different form of identity than ethnic or national groups (Bliuc et al., 2007), but motivates collective action participation more strongly than other forms of group identification (McGarty et al., 2009).

People more readily feel empathy with members of their own group than non-members, and this difference has an impact on behavioral intentions, such as helping (Stürmer et al., 2006). Therefore, ideas of citizenship can increase or decrease empathy and intergroup action intentions by affecting both perceptions of similarity among members and the permeability of group boundaries (for the connection between global citizenship, empathy and intergroup helping see Reysen and Katzarska-Miller, 2013). As we have seen, those who perceive entry into the national ingroup in more flexible ways and consider the nation as an inclusive category may feel more empathy with members of ethnic minorities or immigrants compared to those who define the national ingroup in more rigid and exclusive ways. Therefore, we expect that different definitions of citizenship can elicit higher or lower empathy with members of outgroups, and mediate the connection between definitions of citizenship and intergroup action intentions. However, empathy becomes especially important when injustices are caused by the actions of one’s own advantaged ingroup. It leads people to engage in social change actions to reduce their collective guilt (Calcagno, 2016) and to improve the moral image of their ingroup (Täuber and van Zomeren, 2013). Consequently, in the presence of empathy and injustice awareness, majority group members may be motivated to engage in collective action as allies. Therefore, definitions of citizenship may elicit intergroup action tendencies differently in the presence of high or low empathy with the outgroup, thus functions as a moderator in the relationship as well.

Putting together the results of previous research on national identity, intergroup emotions, and collective action presented in the introduction, we argue that the treatment of disadvantaged ethnic minority groups as well as ethnically different immigrant groups are dependent on ideas about the nation. People who endorse an ethnic definition of citizenship are more likely to feel threatened by ethnic minority and immigrant groups and this threat would motivate engagement in pro-majority collective action. Clearly, empathy with people whose suffering is caused by the ingroup would be hindered by ideas about ingroup superiority among nationalists and that the ingroup can do no wrong. In contrast, people who endorse a civic citizenship can have a more critical awareness of social injustices even if their ingroup is responsible for them, and are therefore more likely to feel empathy for minorities, which in turn motivates them for collective action as allies. These predictions fit into the literature on emotions and specifically on intergroup emotions suggesting that emotions have antecedents and consequences (Iyer and Leach, 2008).

### Research Question and Hypotheses

Our research questions are whether the endorsement of different definitions of the nation predicts pro-social and hostile intergroup behavioral intentions differently, and whether the connection between the definition of the nation and intergroup behavioral intentions is mediated by empathy and threat. Specifically, we hypothesized that the endorsement of an ethnic definition would predict lower pro-minority and higher pro-majority collective action and this connection would be mediated by higher threat and lower empathy. In contrast, we hypothesized that the endorsement of a civic definition would predict higher pro-minority and lower pro-majority collective action and this connection would be mediated by lower fear and higher empathy. However, both threat and empathy can stem from experiences not directly connected to citizenship, yet affect intergroup behavioral intentions. Therefore, these two intergroup emotions can potentially be treated as moderators as well, thus not mediating, just amplifying or weakening the connection between citizenship and action intentions.

As most research related to this issue focused on ethnically different immigrant groups, and not on historical ethnic minorities or specifically the Roma, we did not make specific predictions regarding differences in collective action intentions related to these two outgroups, but generally predicted that the pattern would be identical for the Roma and the immigrant outgroups.

We tested these connections in two studies to establish both the association between the study variables and their causal connections. In Study 1, we conducted an online survey to show the connection between different definitions of citizenship and both pro-minority and pro-majority action, and show whether empathy and fear mediates this connection. In Study 2, we manipulated the concept of citizenship by making an ethnic or a civic definition salient, and tested whether it affected pro-minority or pro-majority collective action tendencies. We also checked whether the connection is different in the presence of high or low empathy and fear. Both studies were conducted following the IRB approval of Eötvös Loránd University.

## Method

### The Context of the Current Studies

The idea of multiculturalism and tolerance has never been adopted in Eastern Europe, and despite the cultural and linguistic diversity of the region, most contemporary nation states are rather homogenous ethnically and endorse ethnic definitions of citizenship (Reijerse et al., 2012). The idea of ethnic citizenship has been central to the current right-wing government of Hungary too. They have held so-called national consultations since 2011 in which they communicated their program and political visions (Government of Hungary, 2017). These national consultations served the purpose of direct public legitimation for the government (see a reflection on one of the national consultations by Bearak, 2017). The first two national consultations are relevant for the current research as they were concerned with defining the members of the national ingroup and its enemies, putting forward an ethnic definition and emphasizing the impermeability of its boundaries by for example items such as these: “There are people who suggest that Hungary’s new constitution should express the value of national belongingness with Hungarians living outside the borders, while others suggest that it is not important” or “There are people who suggest that Hungary’s new constitution should defend our national resources, especially the land and water.” (referring to a ban on foreign ownership).

This social and political context creates a hostile environment for the Roma, the largest ethnic minority group in Hungary. Although Roma people have lived in Hungary since at least the 15th century, they continue to be treated as second class citizens in mainstream political discourse, resulting in institutional discrimination, social marginalization, and poverty (Feischmidt et al., 2013; Kovarek et al., 2017). Their history in Hungary can be characterized by swings between forced assimilation and extreme forms of discrimination starting with their settlement in the 18th century, culminating in the Porrajmos, the Roma Holocaust in the Second World War (Hancock, 2004). Negative stereotypes about the Roma include criminality and laziness, suggesting that their rejection from the majority society is dependent on their lack efforts for becoming accepted members of society (Kende et al., 2017).

The rhetoric of ethnic citizenship was utilized in the anti-immigrant propaganda that started in 2015 (Tremlett and Messing, 2015). This propaganda included different waves of national consultations, media campaigns with messages of threat about illegal Muslim immigration, and a referendum against immigration. Eventually, anti-Muslim and anti-immigrant attitudes exceeded any other form of intergroup hostility in Hungary, including prejudice against the Roma (Simonovits and Bernáth, 2015; Wike et al., 2016). These changes took place despite the fact that Hungary has not been the target of any terrorist attacks of Islamic extremists, nor does it have a significant or visible Muslim or non-ethnic Hungarian immigrant population.

### Study 1

#### Sample

Originally, we relied on a sample of 1080 participants from an online participant pool using a multiple-step, proportionally stratified, probabilistic sampling method resulting in a sample demographically similar to the Hungarian population in terms of age, gender, level of education, and type of settlement. The recruitment was carried out by a professional public opinion company. We did not conduct sample size calculations based on a priori estimations of effect size, but targeted *N* = 1000 that is typically used in opinion poll surveys relying on representative samples of Hungarian society (for the accuracy of estimating election results in Hungary using different sample sizes see Poll of Polls, 2018). Using this sample allowed us to test our hypotheses on an extensive and diverse sample of the Hungarian population. Eleven participants declared that they belonged to the Roma minority. They were removed from the analyses which left us with *N* = 1069. The sample was randomly split: half of the respondents received a questionnaire related to the Roma (*n* = 517), and the other half related to Muslim immigrants (*n* = 552). We used the term Muslim immigrant (“muszlim bevándorló”) throughout the questionnaire to refer to the group of people that represent the most recent wave of immigrants mostly from the Middle East and Africa in order to distinguish this group from a large group of ethnic Hungarian immigrants from neighboring countries. We avoided the term refugee, migrant or illegal immigrant as these terms are heavily politicized in the Hungarian context. However, this group can include non-Muslim immigrants too, such as for example Christians from Syria, therefore we refer to them as immigrants in the paper. The sample consisted of 52.2% women and 47.1% men, and the mean age was 46.8 years (*SD* = 15.67, 18–79). In terms of education level, 34.1% had a higher education degree, 44.1% had secondary education, and 21.9% lower than secondary education; 15.4% lived in the capital city, 54.3% in another city, 29.2% in villages, and 1.1% abroad. All participants were Hungarian nationals. No participants indicated that they were Muslim. On a one to seven scale of self-placement from left-wing to right-wing, the mean score was 4.23 (*SD* = 1.74).

#### Measures

Scales in the questionnaire for the Roma and immigrant outgroups were identical, but slightly rephrased to adapt to their different contexts where it was necessary. Additionally, some attitude scales were presented in both questionnaires and analyzed for the full sample, and variables not related to outgroups were all identical. Data collected for the current study was part of an omnibus survey. Respondents were informed that the survey would consist of questions related to social and political issues. Questions of citizenship were asked immediately after the demographic questions, not preceded by any other scales. Following the question of citizenship, we asked about the approval or disapproval of hostile political discourse against either the Roma or immigrants for the purpose of another study. This may have focused respondents’ attention to the situation of these out-groups within the political climate of the country rather than within other (non-politicized) context that we found favorable for the current study. These questions were followed by the question of empathy and threat. Questions regarding collective action were asked immediately after questions about threat. All other scales were presented following the scales of the current study. We present all variables and data exclusions related to the current research question. Answers were indicated on a 7-point scale (from 1 = completely disagree to 7 = completely agree) on all items, unless otherwise indicated.

For the ethnic and civic perception of citizenship, we relied on the items derived from the scale of the International Social Survey Programme (International Social Survey Programme, 2014) with one item indicating *ethnic citizenship* (“to have Hungarian ancestry”), and one item indicating *civic citizenship* (“to feel Hungarian”) following the original ISSP instruction “Some people say that the following things are important for being truly Hungarian. Others say they are not important. How important do you think each of the following is…”. Complex constructs such as ideas of citizenship are ideally not tested using a single-item scale (Bergkvist, 2015). The ISSP scale may be the most widely used measure of these two forms of citizenship, but the scale does not yield to an acceptable two-factor model in the Hungarian context^1^. Problems with the operationalization of these two forms of citizenship is also supported by critiques suggesting that language and religion may not be so strongly connected to the concept of citizenship (e.g., Reijerse et al., 2013). Therefore, we made a theoretical decision to rely on individual items that best reflected ethnic and civic definitions in the context of Hungary. Ancestry has been shown to be the core component of ethnic citizenship, and “to feel Hungarian” was the strongest component of civic citizenship in the cross-cultural comparative study of Reeskens and Hooghe (2010). Furthermore, “to feel Hungarian” is the most liberal definition of citizenship in the sense that it sets no external limits to gaining entry into the group, but solely relies on individuals’ wish to join the group. Hence, it seemed the most suitable item for testing whether these two approaches to citizenship predict different outcomes. Nevertheless, it must be noted that “to feel Hungarian” may be interpreted in different ways by respondents, as it captures both the affective and the cognitive component of social identity. For example, when choosing this option as an important aspect of belonging to the national ingroup, some people may put an emphasis on self-categorization as Hungarian, while others on the affective commitment to the group (for a distinction see Ellemers et al., 1999). Respect for law and political institutions may be more central to the idea of civic citizenship, this item appears as an expectation within the concept of ethnic citizenship as well, shown by its high cross loading in previous studies (Reeskens and Hooghe, 2010). Although two other items of citizenship were included in the questionnaire (“to be born in Hungary” and “to respect Hungary’s political institutions and laws”), they were not used in the analysis.

We measured *pro-minority collective action* intentions by four items asking participants about their action intentions in case Roma people/Muslim immigrants moved close to their home to make the context of the questions more real. These items were put together following the example of van Zomeren et al. (2012). Respondents could explicitly state intentions to do something against injustices (“I would support actions to protect the rights of Roma people/Muslim immigrants,” “I would stand up against the segregation of Roma people/deportation of Muslim immigrants,” “I would sign a petition to stand up for the rights of Roma people/Muslim immigrants,” “I would encourage my acquaintances to participate in protests for the right of Roma people/Muslim immigrants” Roma: α = 0.87, immigrant: α = 0.76).

*Pro-majority collective action* was measured by four items similar to the pro-minority collective action items, but adapted to the different context [“I would support actions to protect Hungarians from minorities,” “I would participate in a protest for protecting the rights of Hungarians,” “I would encourage my acquaintances to stand up for the rights of Hungarians,” “I would not vote for a party or politician who claims to protect the rights of Hungarians rather than the rights of minorities” (reverse scored)]. Because of low reliability, the reversed item had to be removed from the scale, the remaining three items had good reliabilities (Roma: α = 0.77, immigrant: α = 0.77).

*Empathy* toward the minority groups was measured using a single item asking specifically whether respondents felt empathy toward the groups in the current political context. The item “együttérzés” refers to emotional rather than cognitive empathy, and is used similarly – in some cases even interchangeably – with sympathy or compassion in everyday language. Although cognitive empathy has been identified as more closely connected to intergroup helping behavior while emotional empathy with avoidance (Einolf, 2012), we opted for the use of this term because the word “empátia” (the literal translation and technical term for empathy in Hungarian) is less known outside academia. We measured perceived *threat* based on the integrated threat theory (Stephan and Stephan, 2000), using six items describing both symbolic and realistic threat to tap into general fear related to the minority groups (for example “Rome people/Muslim immigrants pose a health threat to Hungarians,” “The cultural values of Roma people/Muslim immigrants are in opposition with Hungarian values” Roma: α = 0.83, immigrant: α = 0.89, based on Kteily et al., 2015). Although we measured both symbolic and realistic threat regarding both groups, factor analysis revealed only one factor of threat in line with the findings from previous research in the same intergroup context (Kende et al., 2017). Nevertheless, we checked the results entering the two forms of threats as two separate variables in the model that yielded to a similar pattern as the model with one threat variable. The two forms of threat were highly correlated in both samples (Roma *r* = 0.70, *p* < 0.001, immigrant *r* = 0.83, *p* < 0.001). Information on the model is available in the **Supplementary Materials**.

In order to compare attitudes toward the two groups, we used the *feeling thermometer* in both subsamples in connection with the two outgroups, using a 10-point scale (from 1 = very unlikable to 10 = very likeable). We also measured *intergroup distance* using two items, one about perceiving Hungarians and the Roma/Muslim immigrants as one group in Hungary, and a second item about perceiving Hungarians and the Roma/Muslim immigrants as one group in the world (based on Riek et al., 2010, the correlations of the two items for the combined samples were Roma: *r* = 0.78, *p* < 0.001, immigrant: *r* = 0.74, *p* < 0.001).

#### Results

##### Descriptive statistics

Little MCAR test indicated that data was missing at random in all the variables included in the analysis [Roma: χ^2^(36) = 34.65, *p* = 0.533; immigrant: χ^2^(13) = 11.95, *p* = 0.532].

The comparison of attitudes toward the two groups showed a stronger dislike of immigrants than Roma people based on the scores of the feeling thermometer [Roma: *M* = 4.07, *SD* = 2.34, immigrant: *M* = 3.32, *SD* = 2.35, *t*(1018) = 10.87, *p* < 0.001], and a stronger perception of the Roma and Hungarians as one group than of immigrants and Hungarians [Roma: *M* = 4.64, *SD* = 2.23, immigrant: *M* = 3.82, *SD* = 2.77, *t*(1035) = 8.45, *p* < 0.001].

Descriptive statistics and correlations between all study variables are shown on **Table 1**. Correlations between variables suggest that in connection with the Roma outgroup the acceptance (or reversely the rejection) of the *civic* definition was associated with higher empathy, lower threat, higher pro-minority and lower-pro-majority action intentions, while in connection with the immigrant outgroup the rejection (or reversely the acceptance) of the *ethnic* definition showed the same pattern of connections. Empathy and threat were strongly correlated with both types of action intentions in case of both outgroups. Civic and ethnic definitions of citizenship showed weak positive correlations in both samples.

**Table 1:** Means, standard deviations and correlations between all measured variables of Study 1.

	Roma							
	*M*	*SD*	Ethnic	Empathy	Threat	Pro-minority CA	Pro-majority CA	Political orientation
Civic	5.08	1.63	0.21^∗∗^	0.19^∗∗^	−0.13^∗∗^	0.19^∗∗^	0.02	−0.01
Ethnic	4.53	1.64	–	−0.03	0.10^∗^	0.01	0.27^∗∗^	0.11^∗^
Empathy	3.50	1.70		–	−0.58^∗∗^	0.64^∗∗^	−0.45^∗∗^	−0.15^∗^
Threat	3.95	1.24			–	−0.61^∗∗^	0.53^∗∗^	0.21^∗∗^
Pro-minority CA	3.08	1.39				–	−0.35^∗∗^	−0.17^∗∗^
Pro-majority CA	4.15	1.55					–	0.18^∗∗^
Political orientation (left-right)	4.12	1.81						–
**Muslim immigrants**								
Civic	5.18	1.54	0.14^∗∗^	0.11^∗^	−0.03	0.05	0.05	0.10^∗^
Ethnic	4.64	1.64	–	−0.20^∗∗^	−0.34^∗∗^	−0.20^∗∗^	0.39^∗∗^	0.22^∗∗^
Empathy	3.18	1.72		–	−0.64^∗∗^	0.61^∗∗^	−0.47^∗∗^	−0.30^∗∗^
Threat	4.63	1.46			–	−0.66^∗∗^	0.64^∗∗^	0.42^∗∗^
Pro-minority CA	2.79	1.35				–	−0.41^∗∗^	−0.35^∗∗^
Pro-majority CA	4.49	1.52					–	0.40^∗∗^
Political orientation (left-right)	4.31	1.68						–

*^∗^p < 0.05, ^∗∗^p < 0.001.*

##### Hypothesis testing

**FIGURE 1:**
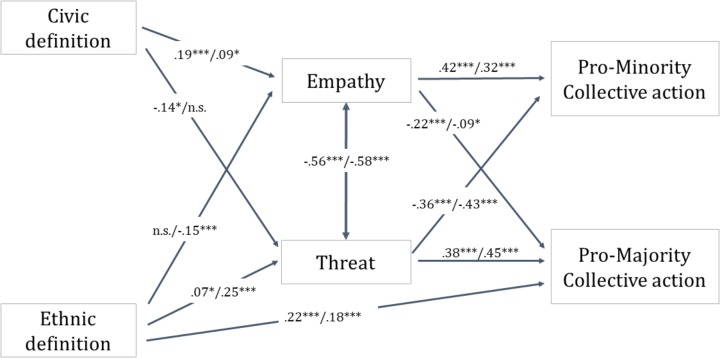
Relationship between variables in Study 1 based on the mediation analysis with political orientation controlled in the model. Numbers represent standardized coefficients for both groups. The first number before the slash refers to the Roma outgroup, and the second to the immigrant outgroup. ^∗^*p* < 0.05, ^∗∗∗^*p* < 0.001.

We used Structural Equation Modeling (using AMOS 22.0) to test our hypothesis that the endorsement of an ethnic definition would predict lower pro-minority and higher pro-majority collective action and that this connection would be mediated by higher fear and lower empathy, while the endorsement of a civic definition would predict the opposite. We included political orientation as a control variable to test whether we can identify predictions beyond the effect of left-right orientation. We identified the most adequate model using the model building – model trimming technique (see Shah et al., 2005). We built a saturated model in which ethnic and civic definitions were allowed to predict both pro-minority and pro-majority collective action mediated by both types of emotions. Such a saturated model shows a perfect fit with χ^2^, RMSEA, and SRMR values of 0, and a CFI value of 1. We then trimmed the non-significant paths to create simultaneously sufficient and parsimonious models that enable us to test the effects of both the civic and the ethnic definition (for a visual presentation of the path with standardized coefficients, see **Figure 1**). The trimmed models still showed very good fit for both outgroups (Roma: χ^2^ = 13.98, *p* = 0.082; *df* = 8; *CFI* = 0.993; *RMSEA* = 0.038; *SRMR* = 0.024; immigrant: χ^2^ = 12.46, *p* = 0.029; *df* = 5; *CFI* = 0.994; *RMSEA* = 0.052; *SRMR* = 0.028).

To reveal whether the relationships between ethnic and civic citizenship on the one hand, and pro-minority and pro-majority on the other hand were mediated by threat and empathy, a series of mediational analyses was conducted with the bootstrapping technique suggested by Macho and Ledermann (2011), where we requested 95% confidence intervals using 5000 resamples. An indirect effect is considered significant if the unstandardized 95% confidence interval around the estimate does not contain 0. Significant indirect effects are shown in **Table 2**.

**Table 2:** Indirect effects of civic vs. ethnic identity on collective action intentions mediated by empathy and threat toward immigrants and the Roma in Study 1.

Target groups	Indirect pathway	*B*	*SE*	95% Confidence Interval	β	*p*
Immigrants	Civic def. → Empathy → Pro-minority CA	0.02	0.01	[0.01,0.05]	0.03	0.004
	Ethnic def. → Threat → Pro-majority CA	0.10	0.02	[0.07,0.15]	0.11	0.001
Roma	Civic def. → Empathy → Pro-minority CA	0.07	0.02	[0.03,0.11]	0.08	0.001
	Ethnic def. → Threat → Pro-majority CA	0.03	0.02	[0.03,0.06]	0.03	0.046

#### Discussion

The weak positive correlation between ethnic and civic definitions of citizenship underlines previous assumptions that the two conceptualizations of citizenship are neither entirely antagonistic nor completely independent, but those who endorse an ethnic definition would also have expectations regarding personal efforts to be citizens (Reeskens and Hooghe, 2010). Nevertheless, all of our hypotheses were confirmed by the data, that is, the endorsement of an ethnic definition predicted lower pro-minority and higher pro-majority collective action and this connection was mediated by higher fear and lower empathy, while the endorsement of a civic definition predicted higher pro-minority and lower pro-majority collective action and this connection was mediated by lower fear and higher empathy. This result is in line with previous research suggesting the importance of the role of defining the nation in inclusive or exclusive ways (see Minescu et al., 2008; Wagner et al., 2012), and the potential consequences for the integration of minorities (Sides and Citrin, 2007; Hochman et al., 2016; Golec de Zavala et al., 2017). As our outcome variable was pro-minority and pro-majority collective action, we were also able to show that these definitions of the nation matter for mobilization, as they predicted intergroup emotions differently which in turn predicted both types of mobilizations in opposite ways.

However, simple correlations and the strength of these connections suggested that different aspects of the definition of the nation were important in connection with the Roma and with immigrant outgroups. In case of the Roma, the endorsement of a civic definition was a stronger predictor of intergroup emotions and collective action, while in the case of immigrants, the ethnic definition was the stronger predictor. The distinction may be explained by several factors related to the differences in the perceptions of the Roma living in Hungary and the perception of immigrants. Our data provided evidence that immigrants were considered more distant from Hungarians than the Roma. This is similar to Parker (2010) finding that domestic outgroups, such as African Americans or Jews were evaluated differently than Arab people based on symbolic patriotism, but not based on blind patriotism. Furthermore, the basis of rejection of immigrants is precisely their intention to gain some form of citizenship, therefore they represent a threat particularly to those who endorse an ethnic definition of citizenship. In contrast, negative stereotypes related to Roma people mostly revolve around their lack of effort for integration and of respect for majority institutions and laws (Kende et al., 2017), which can be interpreted as violations from the perspective of the civic definition of citizenship.

This study provided correlational evidence from a representative survey that ethnic and civic concepts of citizenship predicted pro-minority and pro-majority intergroup action intentions in opposite ways, and the connection was mediated by relevant intergroup emotions. In order to understand whether personal endorsement of these two concepts has a causal effect on mobilizing people for or against minorities, we conducted an experiment in which we manipulated concepts of citizenship through different positive accounts of what it means to be Hungarian. We expected that the civic as opposed to the ethnic manipulation would predict higher pro-minority and lower pro-majority collective action intentions. However, we expected that this effect would be strongest in the presence of high empathy with the minority groups and low fear from them. We therefore tested a moderation effect of intergroup emotions rather than a mediation effect as opposed to Study 1.

This different statistical approach was due to the different designs of the studies and the type of manipulation that we used in Study 2. In the survey we measured people’s pre-existing ideas of the nation that we expected to be the basis of intergroup emotions as previous research suggested (in connection with threat see e.g., Coryn et al., 2004; Sniderman et al., 2004; in connection with empathy see e.g., Thomas et al., 2009; Calcagno, 2016). However, in Study 2 we used positive accounts of the nation to increase the salience of the ethnic and civic aspects of citizenship, respectively, that did not contain any information directly related to minorities. Furthermore, the manipulation was positively framed, and therefore, we did not expect that it would directly elicit empathy toward the groups or fear from them as it could have been in the case of using for example identity threatening manipulations that elicit intergroup emotions (e.g., Hutchison et al., 2006). However, we expected that the positive text of the manipulation would resonate more strongly and increase pro-minority collective action intentions if respondents already had higher empathy toward the outgroups and lower fear from them. In contrast, we expected that the effect of the manipulation would be stronger in the absence of empathy and higher fear from the outgroup. For this reason, we relied on people’s preexisting intergroup emotions as the moderators of the effect of ideas about citizenship.

### Study 2

#### Design

We used an experimental design in which we manipulated definitions of citizenship. In order to increase the personal endorsement of either the ethnic or the civic definitions, we created descriptions that presented Hungarian identity equally positively, yet the civic manipulation described the nation as an inclusive group, suggesting that inclusion was based on individual efforts, and emphasized the shared history of Hungary with other nations, and the ethnic manipulation suggested that the valuable aspects of citizenship were based on ancestry, and emphasized the uniqueness of its history (for a full description of the text and the pictures of the manipulation see the **Supplementary Materials**).

#### Sample

Respondents were recruited from a university class where students participate in research for credit (*N* = 436). The sample consisted of BA and MA students from all faculties of Eötvös Loránd University. Seven people failed the first attention check question which simply asked about the main topic of the text. The second attention check was related to the core aspects of the manipulation, and 23 respondents in the ethnic and 93 in the civic condition chose the wrong option, that is, they either indicated that they had not remembered the answer or chose the option that was valid for the opposite condition. These respondents were removed from the analysis, resulting in an overall sample size of *N* = 320 (civic *n* = 108; ethnic *n* = 181).

Power analysis was conducted based on the correlations from Study 1 that fell between 0.2 and 0.4 regarding the definitions of citizenship and action intentions. Relying on the weaker connection for a more conservative estimation of sample size, G^∗^Power analysis requested *N* = 328 for 95% power to detect an effect size of Cohen’s *d* = 0.4 (Faul et al., 2009). Our sample was therefore sufficient to test the expected connection even after the removal of participants who failed the attention check.

The original sample consisted of 77.1% women and 22.5% men, the reduced sample had 78.2% women and 21.5% men. The mean age of participants was 20.97 years (*SD* = 2.03) in the full sample, and *M* = 21.6 years (*SD* = 1.31) in the reduced sample. Self-placement from left-wing to right-wing had a mean score was 4.10 (*SD* = 1.26) in the full sample, and *M* = 4.02 (*SD* = 2.12) in the reduced sample.

#### Procedure

Using the Qualtrics platform, participants were randomly assigned to either the civic or the ethnic condition, and were informed that the questionnaire consisted of two independent parts: the first one was concerned with the topic of pride, and the second one with intergroup relations. Presenting the purpose of the questionnaire as a study on pride was supposed to increase the positive endorsement of the definition of the nation and mask the real purpose of the questionnaire about the connection between citizenship concepts and intergroup action intentions. Participants were presented with the text of the manipulation that was allegedly from an actor who answered an open question of a magazine about Hungary. The text was accompanied by portraits of four famous Hungarian people (two sportspeople, a scientist, and a singer). We used visual images to increase the priming effect as shown by for example Abraham and Appiah (2006). In the civic condition one of the sportspeople and the singer were foreign-born and gained Hungarian citizenship later in their lives, while the scientist was of Roma origin. In the ethnic condition, all pictures depicted ethnic Hungarians. One picture of an ethnic Hungarian sportsperson was identical in the two conditions.

After the presentation of the text, two attention check questions were asked to establish whether participants paid attention to the text at all, and to the core message of the text, and one question whether they agreed with the message of the text. Attention check questions were introduced so that participants who did not read the text carefully can be removed from the analysis, and agreement was measured in order to assess whether the text was equally acceptable in the two conditions, and relatedly, to identify a potential backfire effect in either of the conditions. Participants then answered the questions about citizenship that served as a manipulation check testing the effectiveness of the manipulation. They were then forwarded to the “next” questionnaire about intergroup relations where questions about intergroup emotions and collective action intentions related to Roma people and immigrants were asked. Scales related to the Roma and immigrants were presented in a randomized order.

#### Measures

We checked the manipulation using the same single items of *ethnic* and *civic* *citizenship* as in Study 1 from the International Social Survey Programme (2014). Answers were indicated on a 7-point scale (from 1 = completely disagree to 7 = completely agree) on all items.

Intergroup *empathy* and *fear* were tested using single items, asking the extent to which respondents felt the listed emotions when they were thinking about the situation of Roma people/Muslim immigrants. Other emotions were listed as fillers in the scale.

*Pro-minority* and *pro-majority collective action* intentions were measured by the same items related to both minorities from Study 1. Pro-minority collective action with four items (Roma: α = 0.89, immigrant: α = 0.92), and pro-majority collective action with 3, however, the reversed item of the scale needed to be removed because of low reliability. The remaining two items were correlated more strongly (Roma: *r* = 0.57, *p* < 0.001; immigrant: *r* = 0.55, *p* < 0.001).

#### Results

##### Manipulation checks

The texts of the manipulation were generally accepted by the respondents, and there were no differences in the level of agreement with the text in the two conditions. We also checked whether agreement with the manipulation was different when testing it on the sample that included people who failed the attention check questions and without them, and found no differences in agreement either in the full sample [Ethnic: *M* = 5.39, *SD* = 1.40, Civic: *M* = 5.61, *SD* = 1.25, *t*(434) = −1.73, *p* = 0.084], or in the reduced sample [Ethnic: *M* = 5.41, *SD* = 1.40, Civic: *M* = 5.63, *SD* = 1.25, *t*(287) = −1.31, *p* = 0.190]. This similar level of agreement suggests that the failed attention check was not likely the result of reactance or the effect of established attitudes, but rather of a lack of attention.

In terms of the direct effect of the manipulation on the concept of citizenship, following the ethnic manipulation, participants agreed more with the idea that citizenship was primarily based on ancestry, that is, with the core idea of ethnic citizenship than following the civic manipulation [Ethnic: *M* = 4.79, *SD* = 1.54, Civic: *M* = 4.25, *SD* = 1.62, *t*(287) = 2.84, *p* = 0.005]. However, we found no differences in the agreement with the statement that citizenship was a matter of feeling Hungarian [Ethnic: *M* = 6.11, *SD* = 1.17, Civic: *M* = 6.05, *SD* = 1.16, *t*(287) = 0.45, *p* = 0.652]. This result suggests that the manipulation was only effective in changing the idea of citizenship in the ethnic dimension and not in the civic dimension. In sum, despite identifying some problems with the manipulation check questions that can suggest both a superficial engagement in the manipulation and the effect of preexisting attitudes, we can conclude that the texts were positively rated and were acceptable in both conditions (shown by the level of agreement), and the manipulation was effective to the extent that it created differences in accepting the ethnic citizenship idea.

##### Descriptive statistics

Means and standard deviations in the two conditions are presented in **Table 3**. Both empathy and fear scores were around the midpoint in connection with both minority groups. However, in contrast to recent public opinion polls that indicated higher hostility toward immigrants than toward Roma (see Wike et al., 2016), we found that empathy with the Roma was lower than with immigrants, while fear from the Roma was higher. These differences were confirmed by paired-sample *t*-tests [Empathy: *t*(288) = 5.02, *p* < 0.001, CI: 0.28, 0.65; Fear: *t*(288) = −3.21, *p* = 0.001, CI: −0.60, −0.14]. Action intentions in favor of both minority groups were rather low, and lower than pro-majority action intentions that were close to the midpoint.

**Table 3:** Means and standard deviations in the two conditions and in total in Study 2.

	Ethnic *M* (*SD*)	Civic *M* (*SD*)	Total *M* (*SD*)
Empathy Roma	3.44 (1.62)	3.83 (1.67)	3.59 (1.64)
Empathy immigrant	3.96 (1.66)	4.22 (1.64)	4.06 (1.65)
Fear Roma	4.39 (1.72)	4.05 (2.00)	4.26 (1.83)
Fear immigrant	3.94 (1.80)	3.80 (1.93)	3.89 (1.85)
Pro-Roma action	2.38 (1.27)	2.87 (1.64)	2.56 (1.44)
Pro-immigrant action	2.31 (1.38)	2.71 (1.63)	2.46 (1.49)
Pro-majority action	3.81 (1.68)	3.62 (1.55)	3.74 (1.63)

##### Hypothesis testing

Respondents in the civic condition showed significantly higher intentions for pro-minority collective action both in connection with the Roma [Ethnic: *M* = 2.37, *SD* = 1.27, Civic: *M* = 2.84, *SD* = 1.67, *t*(184) = −2.69, *p* = 0.008, Levene’s test indicated unequal variances, *F* = 14.65, *p* < 0.001, so degrees of freedom were adjusted from 287 to 184] and in connection with immigrants [Ethnic: *M* = 2.31, *SD* = 1.38, Civic: *M* = 2.71, *SD* = 1.63, *t*(196.5) = −2.10, *p* = 0.037, Levene’s test indicated unequal variances, *F* = 6.09, *p* < 0.001, so degrees of freedom were adjusted from 287 to 196.5]. However, no differences were found in the intentions for pro-majority action [Ethnic: *M* = 3.81, *SD* = 1.68, Civic: *M* = 3.62, *SD* = 1.55, *t*(287) = 0.94, *p* = 0.349].

**FIGURE 2:**
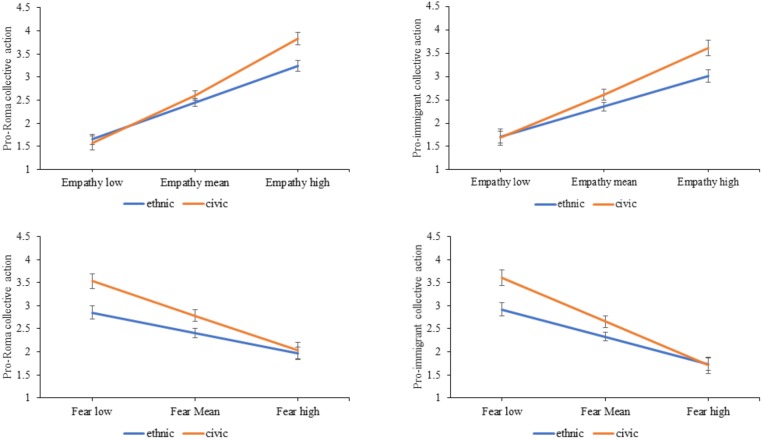
Simple slopes for pro-minority (Roma and immigrant) collective action intentions with empathy and fear as moderators with error bars.

In order to test whether empathy and fear moderated the effect of priming a civic versus an ethnic definition on pro-minority and pro-majority collective action intentions, we conducted a series of general linear regression analyses with an interaction term. Firstly, we ran an analysis with pro-minority collective action intentions as the outcome variable, and entered the interaction of the condition and empathy in the model, then repeated the analysis with the interaction of condition and fear. Secondly, we ran the analyses with pro-majority collective action intentions as the outcome variable, and the same interactions. We found a main effect for empathy on pro-minority collective action intentions [Roma: *F*(1,286) = 44.87, *p* < 0.001, $\eta_{\text{p}}^{2}$ = 0.49; immigrant: *F*(1,289) = 23.64, *p* < 0.001, $\eta_{\text{p}}^{2}$ = 0.36] and fear in opposite directions [Roma: *F*(1,289) = 11.43, *p* < 0.001, $\eta_{\text{p}}^{2}$ = 0.20; immigrant: *F*(1,289) = 16.56, *p* < 0.001, $\eta_{\text{p}}^{2}$ = 0.26], suggesting that participants had higher pro-minority collective action intentions in the presence of higher empathy and lower fear. We also identified a significant interaction effect of empathy on pro-Roma collective actions [*F*(3,285) = 6.72, *p* = 0.010, $\eta_{\text{p}}^{2}$ = 0.02], and a marginal effect on pro-immigrant collective action intentions [*F*(3,285) = 3.15, *p* = 0.077, $\eta_{\text{p}}^{2}$ = 0.01]. Furthermore, fear also moderated the effect of civic vs. ethnic priming on pro-minority collective action intentions for both groups [Roma: *F*(3,285) = 4.04, *p* = 0.045, $\eta_{\text{p}}^{2}$ = 0.01; immigrant: *F*(3,285) = 5.42, *p* = 0.021, $\eta_{\text{p}}^{2}$ = 0.02]. Simple slope analysis with centered empathy and fear variables revealed that respondents in the civic condition indicated higher pro-minority collective action intentions in the presence of high empathy and low fear, that is, pairwise comparisons showed that intentions for pro-minority collective action intentions were only significantly higher in the civic condition compared to the ethnic condition when empathy was high [at +1SD Roma: *F*(1,285) = 10.79, *p* = 0.001, $\eta_{\text{p}}^{2}$ = 0.04, immigrant: *F*(1,285) = 6.77, *p* = 0.013, $\eta_{\text{p}}^{2}$ = 0.02], and fear was average or low [at -1SD Roma: *F*(1,285) = 9.98, *p* = 0.002, $\eta_{\text{p}}^{2}$ = 0.03, immigrant: *F*(1,285) = 10.14, *p* = 0.002, $\eta_{\text{p}}^{2}$ = 0.03]. Simple slopes with error bars for pro-minority collective action intentions with empathy and fear as moderators are presented on **Figure 2**.

Pro-majority collective action intentions were not directly affected by the manipulations, but we still tested whether these intentions were affected differently in the presence of high or low empathy with either minority groups, and high or low fear of either groups. We found a main effect of empathy on pro-majority collective action [Roma: *F*(1,286) = 6.09, *p* < 0.001, $\eta_{\text{p}}^{2}$ = 0.12; immigrant: *F*(1,289) = 5.76, *p* < 0.001, $\eta_{\text{p}}^{2}$ = 0.11], that is, low empathy predicted higher pro-majority collective action intentions. Similarly, fear had a main effect on these intentions [Roma: *F*(1,289) = 4.76, *p* < 0.001, $\eta_{\text{p}}^{2}$ = 0.09; immigrant: *F*(1,289) = 15.94, *p* < 0.001, $\eta_{\text{p}}^{2}$ = 0.25], suggesting that higher fear predicted higher intentions of pro-majority collective action intentions. However, no interaction effect was found with either empathy [Roma: *F*(3,286) = 0.75, *p* = 0.388, $\eta_{\text{p}}^{2}$ < 0.01; immigrant: *F*(3,286) = 0.92, *p* = 0.761, $\eta_{\text{p}}^{2}$ < 0.01] or fear [Roma: *F*(3,286) = 0.48, *p* = 0.488, $\eta_{\text{p}}^{2}$ < 0.01; immigrant: *F*(3,286) = 0.54, *p* = 0.462, $\eta_{\text{p}}^{2}$ < 0.01]. Therefore, the manipulation did not affect those high or low in empathy, or high or low in fear differently.

#### Discussion

Our second study was conducted with the intention to reveal whether different definitions of the nation can have a mobilizing effect on people to become allies of minority groups, or mobilize to protect the ethnic majority group. Our results only partially confirmed this hypothesis, as manipulation check questions suggested that only the ethnic definition was affected by the manipulation and the different definitions of the nation influenced pro-minority action intentions, but did not affect intentions to engage in pro-majority collective action. This is in fact good news for civil rights movements, as even a simple manipulation of presenting the nation in inclusive and civic terms could decrease the idea of ethnic citizenship and increase intentions to participate in collective action on behalf of minorities compared to presenting the nation in exclusive and ethnic terms. In contrast, the findings could be bad news for right-wing nationalist mobilization, as the manipulation was not effective in increasing collective action intentions on behalf of the majority ethnic group. However, this lack of effect also suggests that a more impactful manipulation would be needed to decrease such mobilization intentions.

Overall, our results partially confirmed the causal connection between ideas of citizenship and pro-minority collective action intentions, and clearly indicated that the effect is particularly strong in the presence of empathy and in the absence of fear. These findings reinforce previous understandings of the importance of empathy in ally collective action (see e.g., Thomas et al., 2009), and supplement them by highlighting that fear is not only relevant in intergroup hostility (e.g., Coryn et al., 2004; Sniderman et al., 2004), but its absence is also a precondition for ally collective action intentions. These results indicate that the ethnic definition of citizenship can, to some extent, be manipulated relatively easily, and such an effective manipulation can lead to change in collective action intentions. However, these results also show that interventions that simply make a positive civic rather than a positive ethnic national identity salient can primarily influence people with preexisting empathy with and lack of fear of minority groups. The importance of preexisting attitudes was also highlighted in other studies in which political orientation (Hameiri et al., 2016) and right-wing authoritarianism (Dhont and Van Hiel, 2011) determined how efficient the intervention was in increasing support of intergroup reconciliation and decreasing prejudice. In fact, taking relevant moderators into account seems essential for reaching the full potential of interventions (Walton, 2014).

The lack of effect on pro-majority collective action reveals the limits of such a simple, positive manipulation, and indicates that a more complex manipulation may be needed to influence people’s intentions to join nationalist movements in real life settings. An assessment of positive LGBTQA identity training warns us that even if such an intervention seems efficient in the lab, the effect might not be maintained in the long run as a result of broader, non-supportive societal norms (Riggle et al., 2014). As norms and political rhetoric may have a more substantial impact on the contents of national identities, future investigation on the effects of civic versus ethnic identity manipulation should consider both a stronger manipulation and evaluate its long-term effects.

### General Discussion

With the help of a survey and an experiment, we set out to understand one of the most urgent social issues of Europe and specifically of Hungary, the behavior intentions of members of the majority group in relation to a historical ethnic minority group – the Roma – and immigrants. In Study 1 we identified that civic definition predicted higher pro-minority collective action intentions with regards to the Roma outgroup, and the prediction was mediated by higher empathy and lower threat. In contrast, the ethnic definition of citizenship directly predicted pro-majority collective action intentions, and indirectly predicted both pro-minority and pro-majority action intentions mediated only by threat (in opposite ways, respectively). The same study revealed an almost identical model in connection with the immigrant outgroup, but in this case, the ethnic definition was a stronger predictor of both types of actions, mediated by empathy and threat in opposite ways, but directly predicting pro-majority collective action only. For the immigrant outgroup, the civic definition of citizenship was only a weak indirect predictor of actions through empathy. In Study 2 we tested whether a positively framed manipulation making the ethnic or the civic definition salient can have an effect on action intentions, and found that the civic definition compared to the ethnic one increased respondents’ intention to engage in pro-minority action. The effect was strongest in the presence of higher empathy and lower threat for both outgroups. These two studies provide evidence to the connection between definitions of citizenship and politically antagonistic collective action intentions, and show that empathy and fear play a role in this connection. In Study 1 we found the mediating effect of these two emotions suggesting that a civic definition can predict higher empathy and lower fear that in turn predicts action intentions, and the ethnic definition can predict lower empathy and higher fear that in turn also predicts action intentions. In Study 2 we did not expect that an increased salience of these two definitions would predict higher or lower fear and empathy because the manipulation did not emphasize majority–minority relations directly, but expected and found that the manipulation would be more successful in the presence of intergroup emotions that correspond with the collective action intentions that we measured in our study. While the role of intergroup emotions was conceptualized differently in the two studies, both studies indicated that the connection between definitions of citizenship and collective action intentions is strengthened by empathy and fear.

Previous research on collective action pointed out the importance of group identification (van Zomeren et al., 2008) and intergroup emotions (Thomas et al., 2009), however, definitions of citizenship had not been directly connected to collective action intentions. Bringing together these two lines of research is important both on a theoretical and on a practical level. The theoretical contribution of our research is that we have shown that civic vs. ethnic citizenship ideas are not only important in shaping intergroup attitudes as previous research suggested (Verkuyten and Martinovic, 2015; Mepham and Verkuyten, 2017), but also relevant for intentions to engage in action on behalf of minorities, and to join nationalist, pro-majority movements. These findings bridge the gap between research on citizenship and research on collective action. We have also shown that collective action intentions among advantaged group members with antagonistic political goals can be understood along the same psychological constructs. Furthermore, the similarity of the pattern for both Roma and immigrant outgroups suggests that definitions of citizenship generally affect the treatment of disadvantaged minority groups. Nevertheless, the differences in the strength of the connection indicate that immigrants and historic ethnic minorities are in a different position when it comes to inclusion in the majority ingroup. Ethnic citizenship was more relevant in connection with immigrants, and civic citizenship for the Roma. We assume that this difference has to do with the larger psychological distance with immigrants than with Roma people, and with differences in negative stereotypes connected to the groups.

The practical implications of our research have to do with the nationalist public discourse that is at the core of current right-wing populism (see Mudde, 2004). We have shown that political rhetoric about ethnic definitions of citizenship is associated with nationalist social movements, but it has also highlighted that even with a relatively simple manipulation, civic citizenship can be made salient with consequences for pro-minority collective action. Therefore, our research points out that it could be possible to design effective interventions and show their main direction.

## Limitations and Future Directions

Our research provided both correlational and experimental evidence for the connection between definitions of citizenship and collective action intentions in intergroup contexts. Nevertheless, there are some limitations to these findings. Reflecting on previous debates about the conceptualization of ethnic and civic citizenship (see Reeskens and Hooghe, 2010), and the problems with its operationalization for the Hungarian context, we relied on single-item scales for these constructs. Despite the limitations of single-item measures, they showed good construct validity by predicting the outcome variables in theoretically meaningful ways.

Another caveat is that threat and fear were measured differently in the two studies for solely practical reasons: we used an omnibus survey in Study 1 that already included a measure of threat. However, this measure would have been too long in Study 2 where dependent variables were related to two different outgroups. Such a repetition would have risked respondent fatigue and unreliable responses. Therefore, we opted for shortening those scales that were repeated for both groups in Study 2. As previous research described the intergroup consequences of threat and fear similarly (e.g., Cottrell and Neuberg, 2005), and our research also yielded similar patterns in the two studies, we believe the different operationalization of fear and threat caused no problems for the interpretation of our findings.

We need to interpret the established causal connection between citizenship manipulations and pro-minority collective action intentions with some caution. In Study 1 we created a path model with intergroup emotions as mediators in the connection between the concept of citizenship and action intentions, however, this order of variables does not reflect a causal connection. Indeed, the opposite order of prediction is equally feasible as suggested by Becker et al. (2011) in connection with protest participation and the related emotion of anger. Another caveat of our research in terms of establishing causality was the relatively high and uneven number of failed attention check questions in Study 2. Removal of participants weakened the internal validity of the randomized experiment, as we cannot rule out that those who failed the attention check answered based on their pre-existing attitudes. Furthermore, we used a positively framed manipulation that had a measurable effect only on the ethnic definition of citizenship while it was unable to change the level of the civic definition, suggesting that neither the ethnic framing could decrease the acceptance of the civic definition or the civic definition could increase it. This warns us about the limits of our manipulation of citizenship.

Our first study relied on a large sample that was demographically similar to the Hungarian population, while we used a student pool for our second, experimental study. The relative homogeneity of the sample in Study 2 allowed us to have more control over the effect of the manipulation, but the generalizability of the findings is limited by at least two characteristics of this sample. Firstly, the group consisted of university students, that is young people with higher than average education. Secondly, over 70% of respondents were women. The use of a student sample has well-known limitations (see Sears, 1986), for example their high level of education, and the normative context of the university can create social desirability bias as respondents attempt to appear more open minded and tolerant (An, 2014). This bias may have contributed to the lack of effect on pro-majority collective action intentions that may be seen as non-normative in a university context and especially non-normative among women in comparison with pro-minority collective action. White supremacy movements are often militaristic and represent a masculine culture, and have a disproportionally high male followership (see e.g., Hopkins, 2016; Miller-Idriss, 2017).

Finally, we did not include a control condition in Study 2. We therefore cannot establish whether differences were the result of increasing pro-minority collective action intentions in the civic manipulation or of decreasing it in the ethnic manipulation, or a little bit of both. Understanding which manipulation had the effect would be important for designing interventions. Future studies should therefore extend the design by including a control condition.

Despite these caveats, we provided the first evidence for the direct connection between definitions of citizenship and collective action intentions among ethnic majority group members, and presented the affective processes that contribute to this relationship. We showed this connection with two different outgroups, suggesting general validity while also presenting sources of differences in different intergroup contexts. Finally, our research pointed out that it makes sense to look at the distinct psychological mechanisms of collective action based on intergroup solidarity and nationalist movements simultaneously, as they are both responses to the same political context of growing right-wing nationalism and populism.

The research was complex in the sense that it included two different forms of collective action intentions, two different intergroup contexts, and analyzed two distinct emotional processes. However, this complexity was necessary to explain the common political and psychological roots of politically antagonistic social movements in Europe that our research set out to investigate. Our research has shown the scope and potential of interventions in bringing about social change, and also pointed out the limits of such interventions. Future research should therefore test more effective methods of intervention. In conclusion, we found that discourse about the nation is of vital importance for intergroup relations and for the future of Europe.

## Ethics Statement

This study was carried out in accordance with the recommendations of APA research ethics guidelines, and the institutional (Eötvös Loránd University) and national guidelines. The protocol was approved by the research ethics committee of the Institute of Psychology, Eötvös Loránd University. All subjects gave written informed consent in accordance with the Declaration of Helsinki.

## Data Availability

The datasets generated for this study can be found at osf.io/8wd47.

## Author Contributions

AK contributed the research idea and the general design of the studies. She was responsible for data analysis and the preparation of the manuscript. NL helped with the design of the studies, data analysis, and the preparation of figures and tables. She also contributed to the preparation of the manuscript. PK helped with the design of the questionnaire for Study 1 and helped with the preparation of the manuscript.

## Conflict of Interest Statement

The authors declare that the research was conducted in the absence of any commercial or financial relationships that could be construed as a potential conflict of interest.

## Footnotes

1 Based on calculations made using the publicly available database retrieved from http://www.issp.org, using Maximum Likelihood analysis with Promax rotation we could not identify a two-factor solution, but found three factors with eigenvalues about one. However, religion and feeling Hungarian created single-item scales, while all other scales loaded onto the mixed ethnic-civic factor.
