# Supplementary material for: Endorsing a Civic (vs. an Ethnic) Definition of Citizenship Predicts Higher Pro-minority and Lower Pro-majority Collective Action Intentions
Source: Front Psychol. 2018 Aug 7;9:1402. doi: 10.3389/fpsyg.2018.01402 (PMC6090503; doi:10.3389/fpsyg.2018.01402)
Supplement: Supplementary file 1 [file Data_Sheet_1.PDF]

Supplementary material

An alternative model for Study 1 in which realistic and symbolic threat variables are presented separately. Numbers represent standardized coefficients for both groups. The first number before the slash refers to the Roma outgroup, and the second the immigrant outgroup. *p < .05, *** p < .001.

Model fit indices for the Roma outgroup: χ^2 = 12.45, p = .053; df = 6; CFI = .994; $RMSEA$ = .046
Model fit indices for the immigrant outgroup: χ^2 = 18.29, p = .011; df = 7; CFI = .993; $RMSEA$ = .054

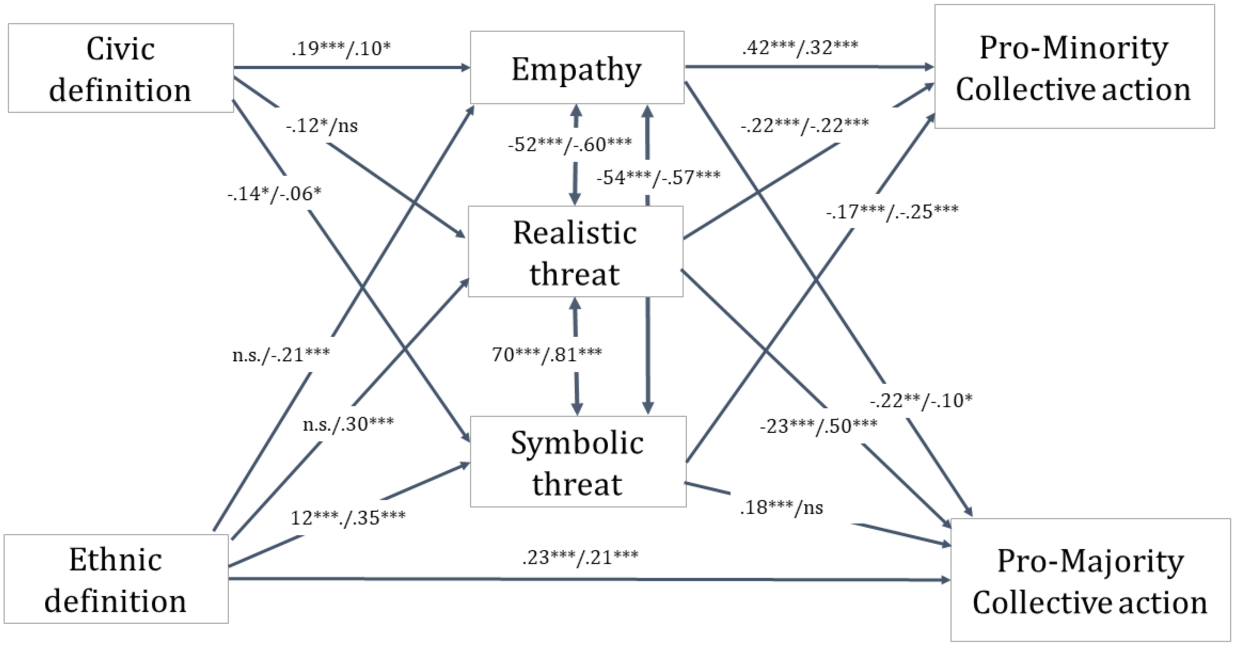

Translated text of the ethnic manipulation and the corresponding pictures in Study 2:

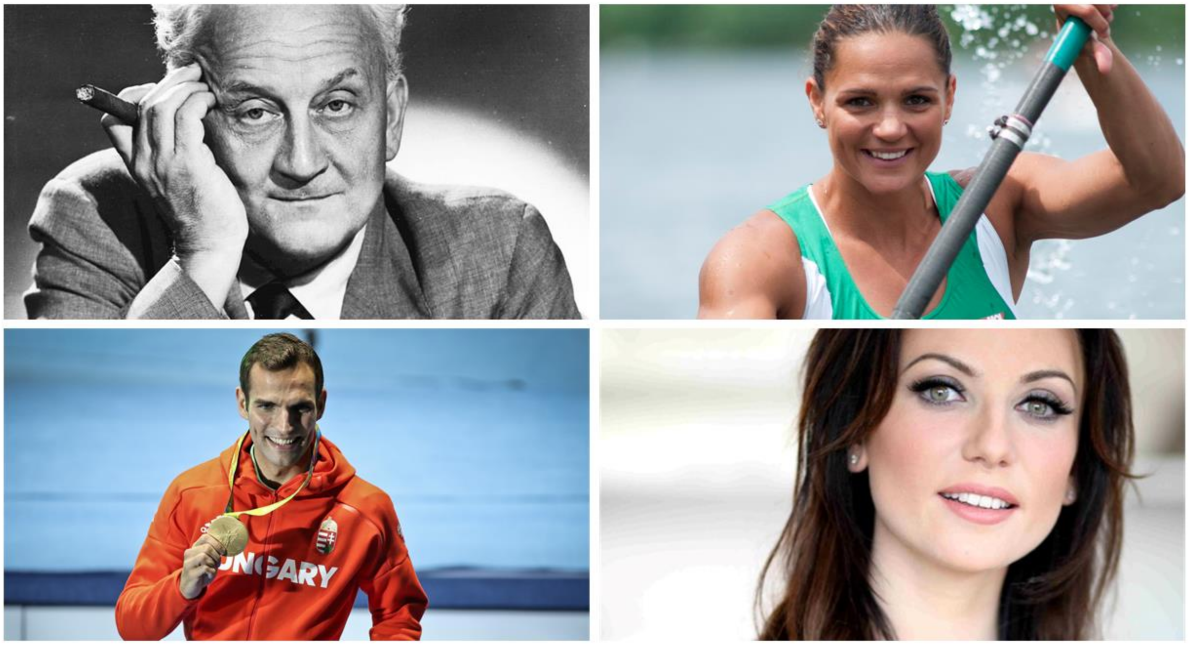

“What is Hungary to me? Hungary is my home. This country can be proud of those compatriots who became world famous as inventors, artists, or sportspeople. They are the great Hungarians. However, we do not only have our successes to share with one another, but we also know that this Central European nation has suffered more under the occupation of powerful empires than any other nation, and because of that we can be even prouder that we maintained our language and culture, and we did not forget what it means to be Hungarian. It does not matter if one is born within the current borders of Hungary or as an ethnic Hungarian outside its borders, the most important thing is that one learns the Hungarian way of thinking, the Hungarian language and culture from their parents and ancestors. This is what Hungary means to me.

Translated text of the civic manipulation and the corresponding picture:

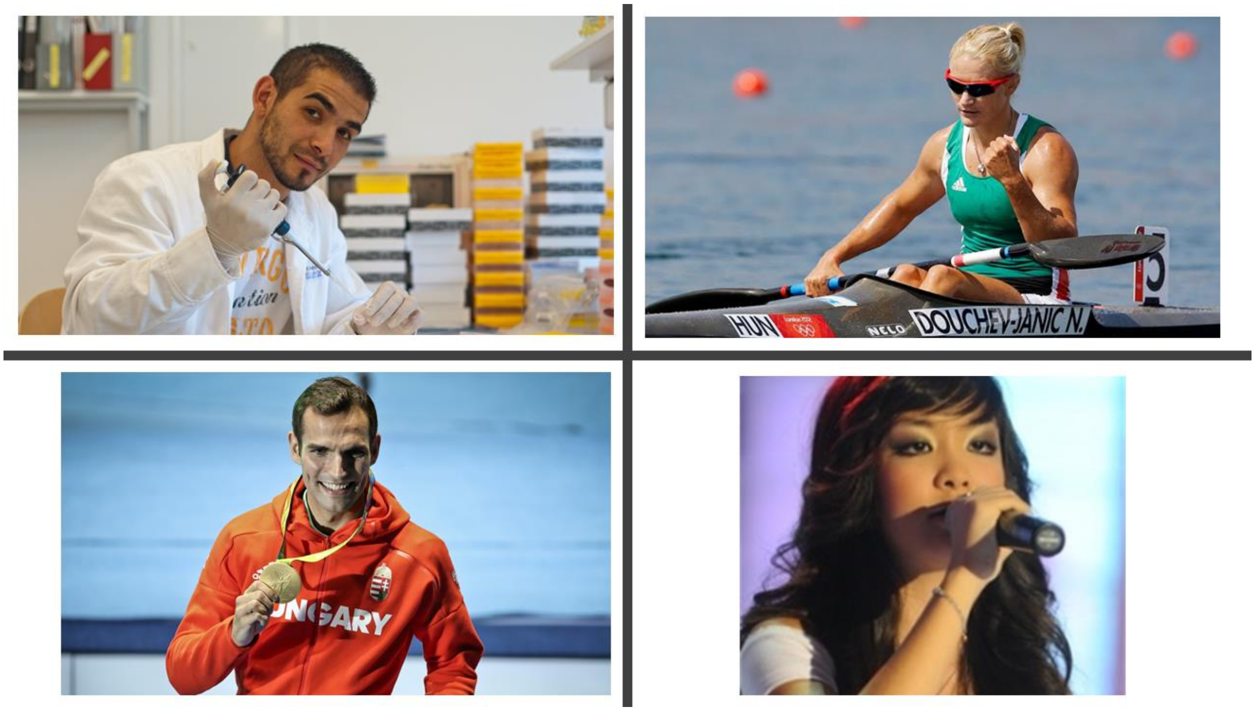

"What is Hungary to me? Hungary is my home. This country can be proud of those citizens who became world famous as inventors, artists, or sportspeople. It does not matter where they were born or what ancestry they have, but that they have done a lot for the country: they are the great Hungarians. However, we do not only have our successes to share with one another, but our historical suffering connects us with other Central European nations as well. We can be proud that we kept our language and culture, and that we did not forget what it means to be Hungarian. It does not matter where one is born or who one's ancestors are, what matters is that we love and care about Hungarian language and culture. This is what Hungary means to me.
